# Detecting bulbar amyotrophic lateral sclerosis (ALS) using automatic acoustic analysis

**DOI:** 10.21203/rs.3.rs-3306951/v1

**Published:** 2023-09-06

**Authors:** Leif Simmatis, Jessica Robin, Michael Spilka, Yana Yunusova

**Affiliations:** University Health Network; Winterlight Labs, Inc.; Winterlight Labs, Inc.; University of Toronto

## Abstract

Home-based speech assessments have the potential to dramatically improve ALS clinical practice and facilitate patient stratification for ALS clinical trials. Acoustic speech analysis has demonstrated the ability to capture a variety of relevant speech motor impairments, but implementation has been hindered by both the nature of lab-based assessments (requiring travel and time for patients) and also by the opacity of some acoustic feature analysis methods. Furthermore, these challenges and others have obscured the ability to distinguish different ALS disease stages/severities. Validation of remote-capable acoustic analysis tools could enable detection of early signs of ALS, and these tools could be deployed to screen and monitor patients without requiring clinic visits. Here, we sought to determine whether acoustic features gathered using a remote-capable assessment app could detect ALS as well as different levels of speech impairment severity resulting from ALS. Speech samples (readings of a standardized, 99-word passage) from 119 ALS patients with varying degrees of disease severity as well as 22 neurologically healthy participants were analyzed, and 53 acoustic features were extracted. Patients were stratified into early and late stages of disease (ALS-early/ALS-E and ALS-late/ALS-L) based on the ALS Functional Ratings Scale - Revised bulbar score (FRS-bulb). Data were analyzed using a sparse Bayesian logistic regression classifier. It was determined that the current relatively small set of acoustic features could distinguish between ALS and controls well (area under receiver operating characteristic curve/AUROC = 0.85), that the ALS-E patients could be separated well from control participants (AUROC = 0.78), and that ALS-E and ALS-L patients could be reasonably separated (AUROC = 0.70). These results highlight the potential for remote acoustic analyses to detect and stratify ALS.

## I. Introduction

Amyotrophic lateral sclerosis (ALS) is an incurable neurodegenerative disease that affects volitional motor control, visceral functions, and cognitive abilities. Survival with ALS, from disease onset, is estimated to be between 20–48 months (Chio et al., 2009). Furthermore, ALS frequently causes speech impairment (Tomik & Guiloff, 2010) secondary to bulbar motor system involvement. This can be devastating for patients and their families and has motivated substantial work to better understand patterns of bulbar/speech changes in people with ALS.

Instrumental lab-based investigations of speech in ALS have demonstrated the value of speech assessment technologies for detecting and tracking ALS progression. The objective measurements afforded by the technologies provide information over and above that which can be gleaned by a clinician (Silbergleit et al., 1997). They can capture early signs of disease (Rong et al., 2015), be used to characterize ALS subgroups, including disease severity classifications (Rowe et al., 2020), and distinguish patients from controls (Vashkevich & Rushkevich, 2021). Detection of early signs of bulbar ALS is a substantial challenge that is very important to address for improving disease management (Goutman et al., 2022). However, lab-based systems tend to be complex and require trained personnel to operate them, even in the context of audio-only recordings. Furthermore, lab-based methods require dedicated lab space and for patients to visit a physical location outside the clinic, which requires time and effort for the patients. This creates barriers to data collection and precludes the incorporation of such tools into clinical practice or clinical trials, ultimately hindering technology adoption.

There has been great interest in developing remote, easy to use, and convenient speech assessment technologies for detection and tracking of ALS progression over time. Remote assessment systems have been developed by several groups in recent years and have demonstrated great promise. For example, they have been used for distinguishing between ALS and control groups (Norel et al., 2018) and quantifying change over time in ALS acoustics (Stegmann et al., 2020). They also have been well-tolerated by ALS patients (Rutkove et al., 2020). Some recent work by Modality.AI has additionally utilized remote assessment for ALS detection as well as stratification of patients into bulbar and presymptomatic (i.e., lacking overt bulbar symptoms) patient groups (Neumann et al., 2021). However, their study focused on only a few features relating to pause timing and rate. There may be additional value in a more representative, but still compact, acoustic feature set that captures speech metrics from other domains such as voice quality. Collectively, an acoustic feature pipeline that can be utilized remotely could be of great value for stratifying patients for e.g., clinical trials or for more effective clinical decision making.

In the present study, we sought to validate an analytical pipeline developed by Winterlight Labs to detect signs of ALS from speech samples, as well as distinguish between severities of ALS-related speech impairments. Winterlight’s remote assessment system has been used extensively for detecting speech and language impairments associated with a variety of neurological and psychiatric diseases (Balagopalan et al., 2019; Fraser et al., 2015; Gumus et al., 2023; Robin et al., 2021), but not yet in ALS. The pipeline extracts a variety of acoustic features making it well suited for ALS assessment. Here, we hypothesized that a core set of acoustic features derived from Winterlight’s assessment pipeline could distinguish bulbar motor stages of the ALS (i.e., AUROC > 0.70) (1) ALS patients from control participants, (2) early ALS (ALS-E) patients from control participants, and (3) early ALS (ALS-E) from late ALS (ALS-L) patients. We additionally hypothesized that (4) weights given to individual features would be clinically interpretable in terms of relation to ALS and disease severity, and (5) that features influenced by sex (e.g., fundamental frequency measures) would not contribute substantially to modelling disease progression.

## II. Methods

Data were collected from 141 participants (119 ALS, 22 controls). See Table I for a summary of relevant clinical and demographic features of the cohorts. For patients only, the ALS Functional Rating Scale-Revised bulbar scale (FRS-bulb) was used to stratify into “early” and “late” bulbar groups using the median value in the dataset, which was 11 out of 12 maximum; i.e., <11/12 is ALS-L and ≥11/12 is ALS-E. Due to missing data for the FRS, n=93 individuals were analyzed when comparing ALS-E vs ALS-L, and n=70 were analyzed when comparing control vs ALS-E. Participants read the Bamboo Passage, which is 99 words in length and assesses various aspects of articulatory and respiratory motor function (Yunusova et al., 2016). Data were recorded in a speech laboratory embedded into a multidisciplinary ALS clinic. The recordings were conducted using a high-quality digital recorder at 44.1 kHz in 16-bit resolution using a cardioid lavalier microphone.

We preprocessed raw acoustic data by removing noise prior to downstream analyses, using Praat (Boersma & Weenink, 2021). At least 0.25 seconds of audio data (i.e., ~10,000 samples) was used for the spectral subtraction noise reduction algorithm (Boll, 1979), with a window length of 0.025 sec, which follows the recommendations on noise reduction in Praat. We selected sample length that was at least several times the length of the window (https://www.fon.hum.uva.nl/praat/manual/Sound__Remove_noise___.html; accessed 7 June 2023). Other settings for noise reduction included suppression range of 80Hz to 10kHz, and 40Hz smoothing. The choice to use lab-based data followed from the purpose of the present study, which was to validate the Winterlight assessment pipeline for both ALS detection and ALS stratification, when data are known to be of high quality and recorded was done under lab conditions.

Further semi-automated quality analysis after noise suppression was performed to ensure high-quality data were analyzed. Thresholds were signal to noise ratio (SNR) > 30Db (Deliyski et al., 2005), clipping in fewer than 1% of data samples (Hansen et al., 2021), and no unusual patterns of noise as evident by visual inspection of spectrograms (e.g., narrowband noise). These steps were performed by trained and experienced research assistants.

Winterlight’s automated pipeline extracts 793 features that encompass various domains of speech and language functioning. For the purposes of the present study, we chose to focus specifically on acoustic features, which we expected to better-reflect the *motor* speech impairment that occurs in ALS. We leave investigation of linguistic features to future work in patients that have more pronounced cognitive deficits, e.g., patients with frontotemporal dementia (FTD) on the ALS-FTD spectrum. Specifically, we focused on a total of 53 acoustic features that reflected the integrity of the respiratory, phonatory, and articulatory speech subsystems. Briefly, these features include, but are not limited to, a variety of speech/pause durations and rates (articulation and respiration), jitter/shimmer/harmonic measures (phonation), as well as additional metrics such as zero-crossing rate. See **Supplementary Table I** for a description of these features in detail. Briefly, feature categories included: jitter/shimmer, fundamental frequency (F0), speech/pause durations, zero-crossings, harmonic/noise ratio (HNR), and intensity.

**Figure 1:**
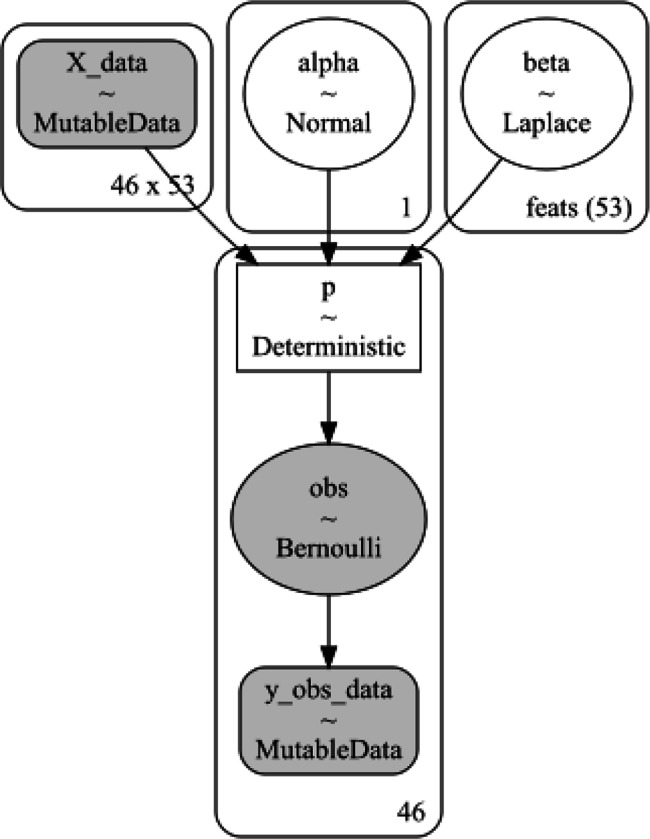
The statistical model used in the present project. Image generated using PyMC v 5.4.1. Alpha is the offset parameter, beta is the vector of *J*=53 regression coefficients (one per feature), and the posterior was modelled as a Bernoulli distribution. *X* and *y* are data objects. Numerical indices reflect the number of individuals. In the figure, the number of participants is 46 (i.e., 50% of the total data in the ALS-E vs ALS-L comparison).

Classification was performed using a Bayesian LASSO (i.e., the Least Absolute Shrinkage and Selection Operator) logistic regression model. See Figure 1 for a schematic diagram of the present statistical model. Following from classical logistic regression, which is a linear operation transformed using a log link function, the present model consists of a global intercept α (i.e., between the two classes being compared at any given time) and a vector of k∈{1…53}β coefficients (i.e., one per acoustic feature). The α parameter was drawn from a standard Normal N(0,1) distribution, whereas the $\beta_{k}$ were drawn from a Laplace L(0.5) distribution, where 0.5 is the parameter controlling the width of the distribution. The latter decision was made to impose a LASSO penalty on the $\beta_{k}$, which is a technique for making coefficients sparse by imposing a penalty on high coefficient values. The Laplace distribution implements this in a Bayesian context (Park & Casella, 2008).

**Figure 2:**
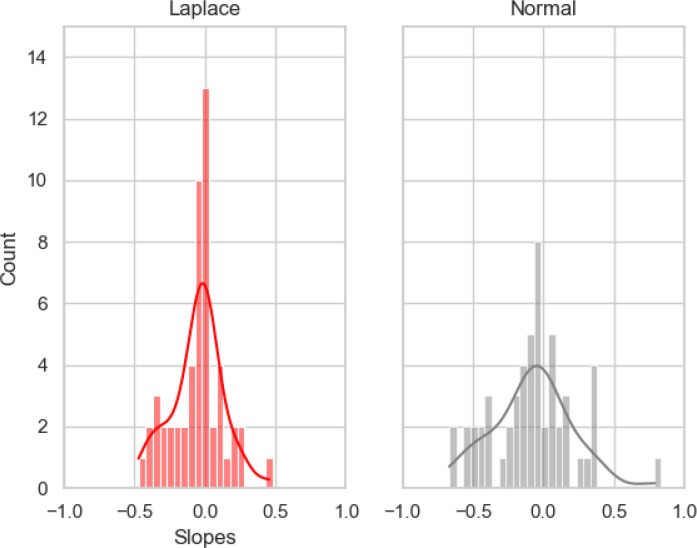
Histograms and kernel density estimates (i.e., the smooth lines) demonstrating the shrinkage of regression coefficients induced by using a Laplace *L*(0.5) prior instead of a Normal *N*(0,1). Data are from one classification fold. Note that a large number of slopes in the Laplace plot are compressed towards zero (indicated by the higher central spike in the left subplot than the right one).

As an example of the parameter shrinkage induced by the LASSO penalty, see Figure 2, which depicts a histogram of parameter values from one of the training folds in the present study, fitted using a Laplace distribution and a Normal distribution. It is evident that the Laplace prior forces parameters to cluster around 0, although retains a number of non-zero parameters. Importantly, this enabled us to more definitively comment on features that had strong impacts on classification decisions, by forcing those with low relative contributions closer to zero.

Binary classifications were performed between: (1) control vs all ALS, (2) control vs ALS-E, and (3) ALS-E vs ALS-L. We performed ten randomized dataset splits, where training data (50%) and testing data (50%) were fully separated. Note that a further split of training into training/validation was not performed, because of the underlying mechanics of the Bayesian model fitting process (there is no hyperparameter tuning as in, e.g., a support vector machine, and so a grid search of hyperparameters is not needed). At each testing iteration, AUROC was evaluated using the predicted score and the ground truth labels. Note that train and test splits were standardized using the mean and variance of the training data.

In addition to binary classification, we investigated the potential contribution of sex as an interactor variable in specific acoustic features, where it would be expected to play a role, given typical differences in vocal physiology between individuals born male and those born female. Specifically, sex effects were modelled in fundamental frequency and HNR features. Interactions were encoded at the data level as multiplicative interactions, and interaction vs no-interaction models were compared using the Watanabe-Akaike information criterion (WAIC).

Finally, the learned β_k_ for each binary comparison and for each classification fold were extracted. The median of these values was calculated for plotting purposes, to provide an indication of the relative contribution of each acoustic feature to each classification decision.

## III. Results

### Classification results

**Figure 3:**
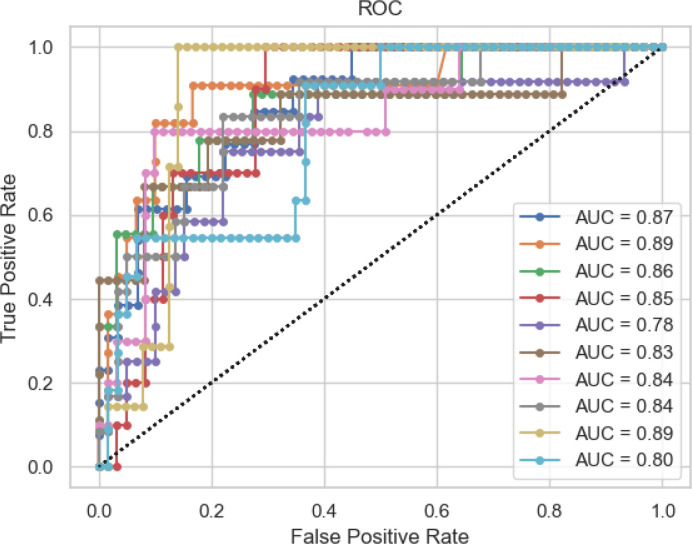
Receiver operating characteristic (ROC) plots for all 10 held-out test set folds of the ALS vs control comparison. AUC (i.e., AUROC) is indicated for each fold. Folds and legend labels for AUC are colour-coded.

Classification results suggested that it was possible to separate ALS and control groups as well as ALS-E and ALS-L; AUROC was ≥0.70 for all comparisons, see Table II. To see a plot of the 10 folds of ALS vs control participants, as an example, see Figure 3. We observed that the mean AUROC of the all-ALS vs control comparison was good (0.85), the AUROC of the ALS-E vs ALS-L comparison was somewhat lower (0.70), and that of the ALS-E vs control comparison split the difference (0.78).

### Feature coefficients

**Figure 4:**
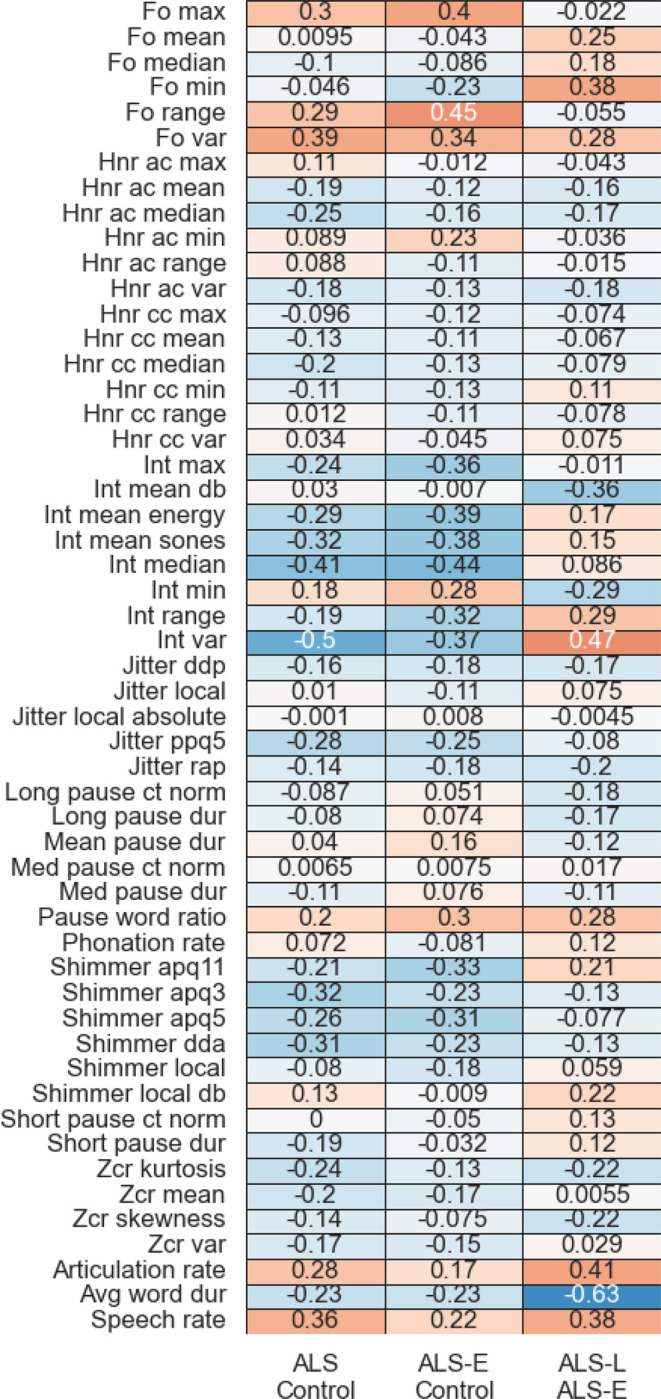
Aggregated feature coefficients across the three different binary classification conditions. In general, positive values correspond to the *less-impaired group having higher values*. Stronger weights are indicated by either darker orange (positive weights) or darker blue (negative weights) cell colours, and weights are additionally annotated. Feature names are indicated on the vertical axis. ac = autocorrelation, apq = amplitude perturbation quotient (3, 5, and 11-point cases), cc = cross-correlation, ct = count, db = decibels, dda = average absolute difference in amplitudes between periods, ddp = difference of difference of periods, dur = duration, Fo = fundamental frequency, HNR = harmonic/noise ratio, int = Intensity, max = maximum, med = medium, min = minimum, norm = normalized, ppq5 = 5-point pitch perturbation quotient, rap = relative average perturbation, var = variance, Zcr = zero-crossing rate.

We identified across the ten train/test splits that certain groups of acoustic features tended to weight more strongly than others. See Figure 4 for a summary of aggregated feature coefficients. It is evident that features from categories such as speaking rate, intensity, F0 distributional characteristics (e.g., range), and shimmer tended to have higher feature weights, whereas ZCR, jitter, HNR, and pause statistics tended to have lower coefficient magnitudes. Some feature weights also reflected differences in disease severity. For example, in the ALS vs control comparison, speech rate had a +0.36 coefficient, indicating that the speech rate in controls was higher than the ALS-E patients. In the ALS-E vs ALS-L comparison, average word duration had a −0.63 coefficient, indicating that the ALS-E average word duration was lower than the ALS-L patients.

### Impact of sex

We observed that the impact of sex as a covariate was not substantial in the majority of the ten models we trained and evaluated. In all cases, the no-interaction model was either a better fit to the data, or the interaction did not fit the data substantially better. Thus, we retained the simpler model without interactions.

## IV. Discussion & Conclusion

In this study, we validated a remote-capable automated pipeline developed by Winterlight Labs for the purposes of stratifying ALS patients by bulbar disease severity. We observed that a relatively small set of the core acoustic features (n=53) derived from the automated analysis were able to detect ALS well (mean AUROC across ten test sets was 0.85) but, importantly, we were able to detect early signs of bulbar impairment at a comparable rate (mean AUROC = 0.78) and could even reasonably distinguish between ALS severities (mean AUROC = 0.70). Furthermore, acoustic features that are known to change with disease severity in ALS (e.g., speech rate) (Yunusova et al., 2016) were given strong coefficients, validating the use of the pipeline for capturing speech changes in ALS. Finally, models that included a sex-interaction term were not substantially better fits for the data than models without interaction terms. These results highlight the substantial promise of the Winterlight system for the detection of ALS as well as the detection of early bulbar changes in ALS patients.

Additional research groups have explored the detection of ALS at various stages using acoustic features (sometimes in combination with kinematic features) and their classifiers’ performance was generally in line with that observed here. Modality.AI (Neumann et al., 2021) used a multimodal dialogue agent to assist in the extraction of acoustic and kinematic speech features. They additionally stratified patients into bulbar and presymptomatic groups. Their AUC performance was comparable to that of the present study; severe patients vs control mean AUC was 0.92, followed by a mean AUC of 0.81 for bulbar vs presymptomatic, and a mean AUC of 0.62 for controls vs presymptomatic patients. Our results by comparison were 0.85 (note: all patients rather than only severe patients), 0.70, and 0.78 for the corresponding comparisons. The difference in performance between the (A) less-severe vs more-severe comparison, and the (B) less-severe vs control comparison may reflect differences in stratification cutoff. Neumann et al did not allow an FRS-Bulb score of 11/12 to count as “early” disease, whereas we did. Additional differences included our preponderance of ALS patients vs controls (119 patients vs 22 controls) compared to the inverse in Neumann’s work (29 patients vs 68 controls). Higher performance of voice-based classification has been reported (Tena et al., 2022; Vashkevich & Rushkevich, 2021), but these either did not stratify patients into groups, or included mel-frequency cepstral coefficients, that we did not include in the present work because of the difficulty in their clinical interpretation.

Salient patterns were observed in the features that were given strong weights in the classification results. For example, rate-related features typically had relatively high coefficient values across all three of the binary comparisons. However, they were much stronger in ALS-L vs ALS-E compared to, e.g., ALS-E vs control. This reflects the greater rate of decline in speaking rate with more advanced disease (Eshghi et al., 2022), although it is notable that (Allison et al., 2017)identified rate/pause related features as important for early detection of bulbar symptoms vs healthy participants; this may reflect differences in the dataset or in the determination of “early” ALS (they used a self-report threshold of <12 on FRS-Bulb, which differs from our present criteria of ≤11/12). Other measures of articulation timing and control such as voice onset time have been shown to differ between early and late stages of ALS as well (Thomas et al., 2022). Additional features from phonatory and respiratory categories may show differential effects of disease severity that could correspond to the findings from the present study. For example, previous work has identified that maximum F0 and F0 range are important features for predicting intelligibility loss (R. D. Kent et al., 1989; Rong et al., 2016), and phonatory instability is known to increase in advanced ALS (Ramig et al., 1990). In terms of respiratory features, previous work has identified that impairment of respiratory muscles (in particular expiratory muscles) occurs rapidly in ALS, which may correspond to the current observation of a strong weight applied to the intensity features (e.g., median intensity) (Heiman-Patterson et al., 2021). Finally, it is notable that many of the features in our models, including the ones aggregated across multiple test-set repetitions, tended to be close to 0. For instance, across all three of the binary classifications, HNR features tended to have low-magnitude coefficient values, suggesting that they were not important for any of the classifications. This is likely a consequence of our choice of regularization approach, which makes interpretation of the patterns across groups more straightforward.

Some of the features that we would have most expected to be affected by sex typically had low feature weights. This was particularly the case in the F0 mean and F0 median features, which had low feature weights in the all-ALS vs control and ALS-E vs control comparisons. This observation supports our choice to not model interactions between sex and acoustic features in the present analysis. We acknowledge that at later stages of disease severity, there can be differential patterns of F0 change between males and females with males demonstrating higher F0 and females – lower F0 with disease progression (J. F. Kent et al., 1992; Robert et al., 1999). This could explain the lower performance for distinguishing ALS severity groups. Notably, the weights for F0 features increased slightly in the ALS-E vs ALS-L comparisons, which is unlikely to be due to a sex imbalance given that in the ALS-L group, 56% of participants were male, compared to 63% in the ALS-E group. Potentially this is due to changes in F0 that occur with disease progression (Ramig et al., 1990).

Our study has some limitations to be addressed in future research. An important consideration is that, of the >750 features in the Winterlight pipeline, only the 53 here pertain to acoustics, and are drawn from a relatively small number of feature domains (e.g., many related to rate and pauses). This limits the ability to represent impairments in diverse domains of speech such as the resonatory subsystem, which can be affected in ALS (Eshghi et al., 2021). It is also notable that the balance of feature types in the Winterlight feature set was biased substantially towards articulatory features. Many of these features were given relatively high feature weights, but they likely captured the same overall constructs, despite measuring e.g., pauses of different durations. Potentially, methods to address collinearity could be of benefit to this analysis in future studies. Classification analysis demonstrated that in cases where the number of features exceeds the number of observations, it is not possible to select more predictors than observations using a Laplace prior(van Erp et al., 2019). In practice, we did not expect to have a large number of important predictors, and empirically we observed good performance of the LASSO (i.e., Laplace) as implemented here. However, this is taken under advisory for future work to explore different coefficient shrinkage methods, particularly in cases where there may be many more than 53 acoustic features to analyze. We also had a relatively imbalanced dataset between ALS and control participants; we addressed this using robust analysis and scoring methods (Bayesian methods with AUROC evaluation) that is resistant to influence by class imbalances. However, a larger control dataset might enable a more detailed appreciation of acoustic patterns associated with healthy performance and enable comparisons between ALS and other neurodegenerative diseases as well. Finally, we explored comparisons between ALS severities and controls or each other in a binary fashion; this led to interesting results and highlighted some interesting patterns in the data; however, a more interesting comparison would be to perform a three-way classification. We did not perform this analysis in the present study because we had a relatively mild cohort overall; future work should explore multiclass classification.

The results of the present study suggest that automated acoustic analysis using a remote-capable pipeline by Winterlight Labs can detect bulbar ALS, as well as earlier stages of the disease. These results show that even with a relatively small set of acoustic features, the Winterlight pipeline could stratify ALS patients into early and late bulbar stages, with clinically-interpretable feature importances. Future work will evaluate detection using more participants and across a greater range of severities.

## Figures and Tables

**Table I:** Smmary of demographic and clinical information.

	ALS	Control
N (num. F)	119 (47)	22 (12)
Age (median [IQR])	59 [14]	52 [8]
FRS-bulbar (median [IQR])	11 [3]	-

All values are formatted as median ± interquartile range.

**Table II:** Classification AUROCs

	ALS/control	ALS-E/control	ALS-E/ALS-L
Group	0.85 ± 0.03	0.78 ± 0.04	0.70 ± 0.04

All values are median ± interquartile range.

## Data Availability

The Research Ethics Board and Legal Services at Sunnybrook Health Sciences Centre prohibit access to data without a data sharing agreement between the authors and the interested investigator(s). Please contact Dr. Yana Yunusova to set up an agreement to access the data used in this paper.
